# Poly(lactic acid)-poly(ethylene glycol)/Magnesium Silicate Membrane for Methylene Blue Removal: Adsorption Behavior, Mechanism, Ionic Strength and Reusability Studies

**DOI:** 10.3390/membranes12020198

**Published:** 2022-02-09

**Authors:** Norilyani Izzati Hasanuddin, Wan Nur Aini Wan Mokhtar, Rizafizah Othaman, Farah Hannan Anuar

**Affiliations:** 1 Department of Chemical Sciences, Faculty of Science and Technology, Universiti Kebangsaan Malaysia, Bangi 43600, Selangor, Malaysia; yanizatie57@gmail.com (N.I.H.); wannurainiwm@ukm.edu.my (W.N.A.W.M.); rizafizah@ukm.edu.my (R.O.); 2 Polymer Research Center (PORCE), Faculty of Science and Technology, Universiti Kebangsaan Malaysia, Bangi 43600, Selangor, Malaysia

**Keywords:** adsorption, magnesium silicate, methylene blue, poly(lactic acid), response surface methodology

## Abstract

In this work, the effect of magnesium silicate (MgSiO_3_) as a filler on poly(lactic acid)-poly(ethylene glycol) (PLA-PEG) membrane was investigated towards the enhancement of adsorption capacity for removal of cationic dye. The preparation and fabrication of membranes were performed through copolymerization and phase inversion techniques. Analysis of functional groups, tensile strength, morphology and surface wettability were employed in the characterization of the membranes. After the addition of MgSiO_3_, it was found that the PLA-PEG/MgSiO_3_ membrane presented a higher hydrophilic property with improved mechanical strength. Next, the adsorption of methylene blue (MB) was optimized using response surface methodology (RSM) with the parameters mass of membrane and initial concentration of MB solution. The effects of pH and ionic strength were also examined to determine the mechanism involved during adsorption processes, which later were found to be electrostatic interaction and ion-exchange mechanism. From the isotherms and kinetics studies, the PLA-PEG/MgSiO_3_ membrane was well fitted by the Freundlich model and pseudo second order model, respectively. This membrane also demonstrated reusable character of up to six cycles.

## 1. Introduction

In a year, almost 1000–3000 m^3^ of effluent gets discharged from the textiles industry per day and approximately 15% of the dyes get washed out due to inefficient dyeing process during manufacturing and processing operations [1,2]. These industrial effluents are comprised of water, dyes and chemicals, some of which are non-biodegradable and synthetic in nature. Dyes have at least one chromophore (color bearing group) and auxochromes (color inducers) which contribute towards coloring, decorative, aesthetic and artistic effects. Due to these effects, the use of dyes became important and one of the main attractions that enhance the commercial value of a particular fabric. However, dyes themselves are toxic, have poor biodegradability, high color depth, are able to resist some action of oxidizing agents and can interfere with light transmission, all of which pose a serious threat to the ecosystem [3,4]. The most commonly used dye in the wood, cotton and textiles industries is methylene blue (MB) [5]. MB belongs to a class of synthetic cationic thiazine dyes. Even at low concentrations, MB can produce a slightly toxic effect that can result in nausea, vomiting, Heinz body formation and methemoglobinemia [6,7,8]. Hence, it is vital to treat this dye in wastewater in an economical and efficient method.

Intensive reviews in removing heavy metals and dyes were reported by previous studies [9,10,11]. However, among all treatment methods of dye removal such as adsorption, coagulation, chemical precipitation, flocculation and membrane filtration [12], there has been little discussion on the combination of the membrane and adsorption methods. A membrane is a physical separation that forms a barrier for matter transport by allowing only certain particles, smaller than the pore size, to pass through [13]. In the case of textile wastewater, the membrane method is only able to separate the suspended solid, and not the dyes, because most of the dyes are water soluble [14]. Thus, the most suitable and simple method to remove suspended solid matters and dyes is through using the membrane-adsorption technique which involves dye molecules to adsorb to the membrane surface while the solid suspension is separated. 

Recently, the incorporation of magnesium silicate (MgSiO_3_) into membranes and composites received enormous attention from researchers across the world. MgSiO3 helps in enhancing the adsorption capacity of membranes with its own properties, namely fast rate of adsorption and high adsorption capacity in addition to improving the physical characteristics of membranes. It was revealed that pure MgSiO_3_ had outstanding adsorption capacities of 244, 107.8 and 418.4 mg/g for Rhodamine B, Cadmium and MB removal, respectively [15,16]. The impregnation of MgSiO_3_ into palm-shell waste powdered activated carbon increased the lead removal from 391.3 mg/g to 419.9 mg/g [17]. Furthermore, MgSiO_3_ has a negatively charged surface that potentially can adsorb cationic dyes due to their large surface areas and richness in silicates groups. This surface charge plays an important role by attracting the cationic dye to the surface during the adsorption process [18,19].

Traditionally, the influence of parameters in the adsorption performance was evaluated one factor at time while keeping other factors at a constant level. This specific technique is known as one-variable-at-a-time [20]. However, this technique has a few major drawbacks which are that it fails to include the complete interaction effects between the parameters as well as requires a large number of experiments. With the use of response surface methodology (RSM), this problem can be resolved by designing a set of experiments. RSM is a collection of mathematical and statistical techniques that are suitable for analyzing the contribution of parameters by determining the interaction between the parameters and modeling the system [21]. The most common and efficient designs used in RSM modeling are Box–Behnken designs (BBD) and central composite design (CCD) [22]. In this study, CCD was chosen because it gives higher prediction of the response while saving time and costs by reducing the number of trials. This design was also suitable for use for at least two or more parameters involved in the experiment.

Poly(Lactic Acid) (PLA) was chosen as the membrane matrix due to its eco-friendly polymer, thermoplastic nature and high strength. Unfortunately, its major drawbacks are that it is hydrophobic and brittle and has poor toughness [23]. Therefore, an excellent plasticizer such as poly(ethylene glycol) (PEG) is needed to improve the matrix membrane, as PEG is a hydrophilic polymer, non-toxic and has good biocompatibility. In addition, PLA co-polymerized with PEG can enhance their membrane’s properties by increasing the hydrophilicity, thermal stability and mechanical strength [24]. Herein, we have successfully fabricated a PLA-PEG membrane and PLA-PEG with magnesium silicate (MgSiO_3_) (PLA-PEG/MgSiO_3_) to evaluate its dual functions which act as filter and adsorbent. The adsorption performance towards MB dye was systematically investigated with the aid of RSM. RSM was used to determine the optimization condition of MB removal efficiency in synthetic wastewater which included mass of membranes and initial concentration of MB solution. Then, the effect of pH solution and ionic strength as well as adsorption kinetics and adsorption isotherms were distinctly evaluated to determine the adsorption behavior and mechanism involved during the adsorption process. Due to the good mechanical properties of the membrane, it was possible to carry out the reusability study of the PLA-PEG/MgSiO_3_ membrane accordingly.

## 2. Materials and Methods

### 2.1. Materials

Poly(lactic acid) (PLA) was acquired from Shenzhen Esun Industrial Co., Ltd. (Shenzen, China) and magnesium silicate was purchased from Dallas Group of America Inc. (Whitehouse, NJ, USA). Other chemicals used were poly(ethylene glycol) (PEG, Sigma Aldrich, Saint Louis, MO, USA) with an average molecular weight of 10,000, tin(II) 2-ethylhexanoate (Sn(oct)_2_, 95.0%, Sigma Aldrich), 1,6-hexamethylene diisocyanate (HMDI, 98.0%, Sigma Aldrich), dichloromethane (DCM, 98.0%, Sigma Aldrich), and tetrahydrofuran (THF, 99.8%, Sigma Aldrich). All chemicals and reagents were used without further purification. Magnesium silicate gel was sieved to the size of 0–45 μm prior to usage.

### 2.2. Preparation of Membranes

PLA (5 g) pellets were dissolved in a round bottom flask containing 30 mL of DCM for 2 h. In the meantime, 0.6 g of magnesium silicate (MgSiO_3_) was stirred with 30 mL THF in a beaker. After a few hours, 5 g of PEG was added into the flask containing PLA solution, followed by 36 mg of Sn (Oct)_2_ and 0.7 g of HMDI. The mixture was stirred for an additional 2 h at 45 °C. Then, the MgSiO_3_/THF mixture was added into the flask containing PLA-PEG copolymer solution and stirred overnight at 45 °C. PLA-PEG/MgSiO_3_ solution was cast onto a glass plate using a casting knife. The solvent was evaporated for 200 s before being immersed in distilled waste and the off-white membrane formed was carefully peeled off. The experiment was repeated in the absence of MgSiO_3._

### 2.3. Characterization of Membranes

Fourier-Transform infrared (FTIR) was used to determine the presence of functional groups in the membranes in the range of 650–4000 cm^−1^. This analysis was run using Perkin Elmer (Waltham, MA, USA) model Spectrum 400, in ATR-FTIR mode. Tensile tests were performed according to the standard ASTM D882 by using an Instron (Norwood, MA, USA) model 5566 universal tensile machine. Several samples of each membrane were tested, and the average values were taken from eight readings. The membranes’ surface morphologies were investigated using scanning electron microscopy (Zeiss/SUPRA 55VP, Oberkochen, Germany) using gold coating. The water contact angle analysis of the membranes was conducted using a drop shape analyzer (Krüss GmbH, Hamburg, Germany, model FM40Mk2). Four microliters of deionized water were dropped on the membrane surfaces and 5 spots of each membrane were taken every 10 ms for 1 min. The average values were calculated.

### 2.4. Adsorption Studies

Each part of the designed experiment was carried out in 50 mL of MB solution (using distilled water, pH = 6.65) before being left at room temperature for 24 h. The equilibrium concentration of MB solution was measured using UV-Visible Spectrometer (Shimadzu Europe/UV-1650 PC, Shimadzu Europe, Duisburg, Germany) at the λ_max_ value at 664 nm. The amount of the MB adsorbed onto PLA-PEG and PLA-PEG/MgSiO_3_ membranes was determined by the difference between the initial and equilibrium concentration of MB solution. The adsorption performance or removal percentage of MB on membranes was calculated using the following equation:

(1) $$R\%=\frac{\left(\mathrm{Co}-\mathrm{Ce}\right)}{\mathrm{Co}}\times 100\%$$

where Co and Ce are the initial and equilibrium concentrations of solutions (mg/L), respectively. The effect of pH solution and ionic strength on adsorption performance was also examined by a fixed amount of 3 mg of porous film and 5.5 ppm of initial concentration of MB under the same conditions and the percentage removal was also determined by using Equation (1).

### 2.5. Response Surface Methodology

Adsorption performance was evaluated by 13 batch experiments designed by central composite design (CCRD) of RSM using Design Expert 7.15 software (Stat Ease Inc., Minneapolis, MN, USA). It is to investigate the effect of mass of membranes (1–5 mg) and initial concentration of methylene blue solution (1–10 ppm). All parameters in CCD were studied in five levels as shown in Table 1 which includes coded –α, −1, 0, +1 and +α. The level coded of α was made by the choice to enable the rotatable design of CCRD. Moreover, CCD design is useful in predicting the linear and quadratic interaction effect for the parameters. It is also said to be more accurate in determining the optimum condition over other factorial designs because of the axial central point data. By using 2 parameters, one set of 13 experimental tests were given with 2^2^ factorial points, the 4 axial points, and the 5 central points which can be expressed as follows:

Total Number of Experiments = 2^k^ + 2k + C_o_ (2)

where k is the number of factors, and C_o_ is the number of central points [25]. Data from these 5 central points are used to determine the reliability of the data and calculate the experimental error [26]. In addition, this model is further improved as compared to Box–Behnken model due to more data points used for optimization calculation.

### 2.6. Reusability and Regeneration Study

**Figure 1:**
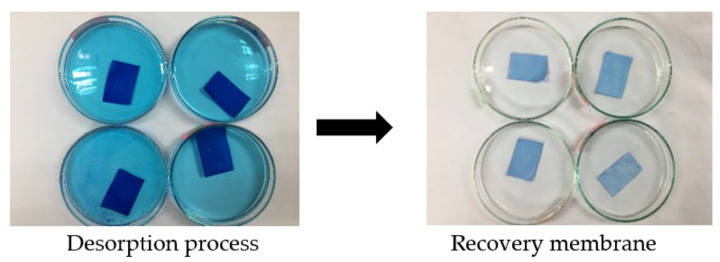
Desorption-adsorption method of membrane.

The reusability and regeneration study was carried out by using methanol as desorbing agent through adsorption-desorption method (Figure 1). After the adsorption process took place, MB-loaded membrane was dried in the desiccator for 24 h. Then, the membrane was immersed in 50 mL of methanol solution for another 48 h. This cycle was repeated upon membrane recovery. After the required time of the experiments, the amount of dye adsorbed for every cycle was measured by using UV-Visible Spectrometer.

## 3. Results and Discussion

### 3.1. Characterization of PLA-PEG and PLA-PEG/MgSiO_3_ Membranes

#### 3.1.1. FTIR Analysis

**Figure 2:**
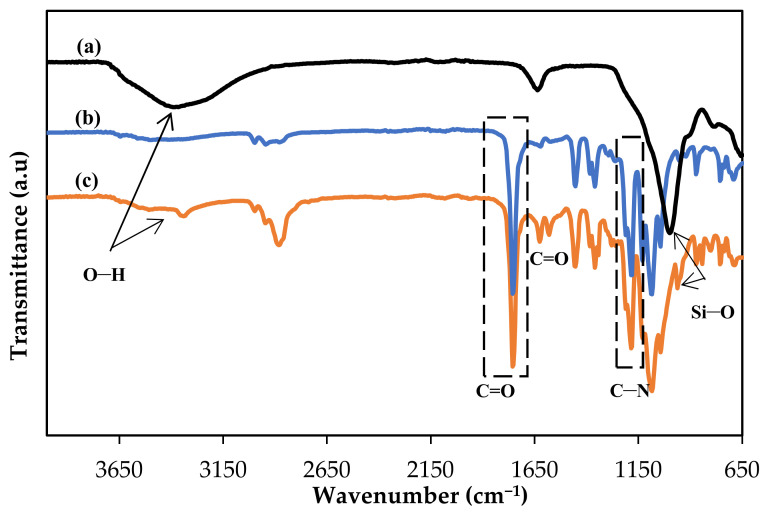
Fourier-transform infrared (FTIR) spectra for (a) MgSiO_3_, (b) PLA-PEG and (c) PLA-PEG/MgSiO_3_ membrane.

FTIR analysis was performed to verify the formation of copolymerization of PLA-PEG with the assistance of chain extender agent, HMDI. This can be proven through the presence of urethane functional groups (–NHCOO). In addition, it is also used to confirm the incorporation of MgSiO_3_ into the PLA-PEG matrix. Figure 2 shows the FTIR spectra for (a) MgSiO_3_, (b) PLA-PEG membrane and (c) PLA-PEG/MgSiO_3_ membrane. In the spectrum of MgSiO_3_, four main bands observed at 3369 cm^−1^ and 1628 cm^−1^ belonged to O–H stretching and bending from Mg–OH while 995 cm^−1^ and 776 cm^−1^ corresponded to the stretching and bending of Si–O [27]. As observed in the spectrum of PLA-PEG membrane, the bands formed at 2952 cm^−1^ and 2933 cm^−1^ were represented by CH_2_ and CH_3_ stretching, respectively [28]. Next, a band with a sharp intensity around 1755 cm^−1^ is attributed to functional group of C=O from pristine PLA [29,30]. This particular band remained unchanged even after the copolymerization of PLA-PEG because during the copolymerization process, only the O–H group from the polymer reacted with the –NCO group from HMDI. The two absorption bands observed around 1617 cm^−1^ and 1181 cm^−1^ were attributed to the stretching of C=O ester and C–N from –NHCOO, respectively [29]. These bands obtained signify that the reaction between the O–H groups from PLA and PEG with –NCO groups from HMDI has successfully occurred. In short, PLA-PEG/MgSiO_3_ membrane exhibited all the bands present in spectra of MgSiO_3_ and PLA-PEG membrane, thus confirming the functionalization of copolymer PLA-PEG and the incorporation of MgSiO_3_ into the matrix membrane.

#### 3.1.2. Mechanical Properties

**Figure 3:**
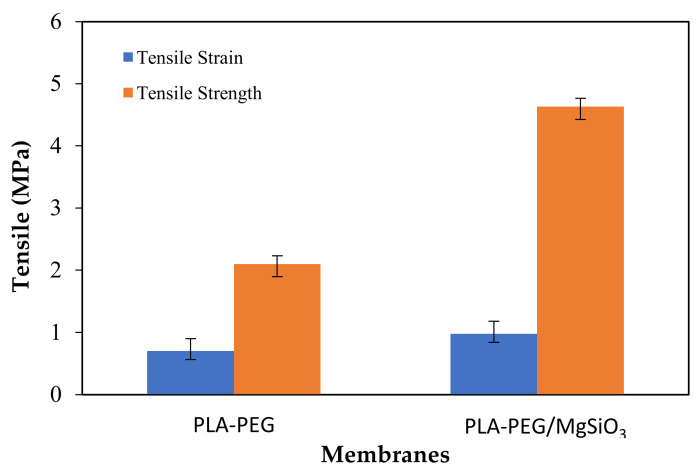
Mechanical properties of PLA-PEG and PLA-PEG/MgSiO_3_ membranes.

The mechanical properties of PLA-PEG and PLA-PEG/MgSiO_3_ membranes were investigated through tensile strength and tensile strain. Figure 3 summarizes the mechanical properties of PLA-PEG and PLA-PEG membranes upon the addition of MgSiO_3_. Based on the results summarized in Figure 3, adding MgSiO_3_ into the membrane’s matrix provides a higher tensile strength and tensile strain, which were 2.1 MPa and 4.6 MPa, respectively, several times higher as compared to the PLA-PEG membrane (0.7 MPa and 0.9 MPa). The increment of mechanical properties of the PLA-PEG/MgSiO_3_ membrane could be related to the filler reinforcement and strong interfacial adhesion between the PLA-PEG matrix and MgSiO_3_. This is because MgSiO_3_ has a large surface area that eventually helps in the better transfer of stress through the shear mechanism from the membrane matrix to the filler, thus resulting in effective load transfer [31,32]. In addition, an improvement in the interfacial adhesion between the PLA-PEG matrix and MgSiO_3_, as can be observed in the next morphology section, is also effective in transferring the stress during breaking, as well as propagation of cracks [33]. It is interesting to note that the strengthening of mechanical properties may also be caused by the interaction ensued in the PLA-PEG/MgSiO_3_ membrane where a hydrogen and covalent bond could be formed, with the assistance of hydroxyl and silane functional groups, between membrane matrix and filler [34]. This finding can be supported by a previous report which demonstrated that the increased amount of filler in a membrane may enhance the tensile properties of the material [35]. To reiterate, the addition of MgSiO_3_ can greatly improve the mechanical properties of the membrane.

#### 3.1.3. Membranes Morphology

**Figure 4:**
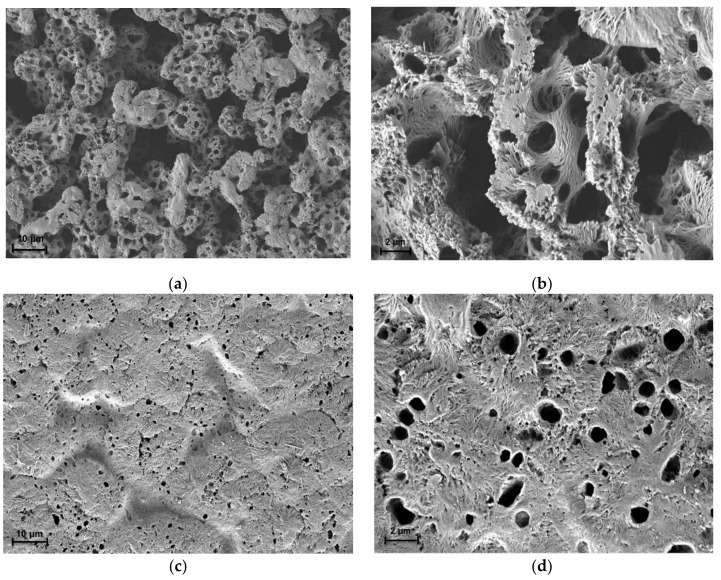
Surface morphologies of (**a**) PLA-PEG at 1000× magnification, (**b**) PLA-PEG at 5000× magnification, (**c**) PLA-PEG/MgSiO_3_ at 1000× magnification and (**d**) PLA-PEG/MgSiO_3_ at 5000× magnification.

To obtain more information about the membranes’ porous structures, the morphologies of both membranes were explored using SEM micrographs analysis. Figure 4 displays the SEM micrographs of PLA-PEG and PLA-PEG/MgSiO_3_ membranes. Both membranes presented a good porosity that was due to the usage of THF solvent and the phase-inversion technique. During phase-inversion, the solvent was exchanged with water molecules in the membrane matrix and became a pore-template after the drying process. In a detailed fashion, Figure 4a shows a rough surface with non-uniform and large pore size in the range of 16 µm to 1 µm as compared to Figure 4c (3–0.6 µm), which could explain a low value in tensile strength performance. Membranes with non-uniform and large pore size tend to be brittle and break easily. Apart from this, the addition of MgSiO_3_ influenced the morphology of the membrane by showing a homogenous surface and smaller pore sizes, as observed in Figure 4c. The filler tends to fill a greater area by being able to occupy spaces in the polymeric matrix [36]. Clear evidence can be seen in Figure 4d, which shows a good interfacial adhesion between the PLA-PEG matrix and MgSiO_3_. This improved interfacial adhesion was the main reason behind the increase of mechanical properties of the PLA-PEG/MgSiO_3_ membrane. This factor is also important as it displayed a major contribution to the adsorption process by regulating the number and availability of adsorption active sites for dye molecules to embed. It is consistent with findings that a smaller pore size of membrane provides a greater surface area for the adsorption process to occur [37].

#### 3.1.4. Surface Contact Angle

Surface contact angle was used to examine the hydrophilicity and wetting behavior of the membranes. Hydrophilicity plays a major role in membranes as it is related to permeation, fouling properties and better wettability. In this study, hydrophilic membranes were desired in order to be applied for wastewater treatment. Membranes exhibited a hydrophilic character once the contact angle became lower than 90°, as the water molecules have a great tendency to wet the membrane surface. The highly hydrophilic and large surface area of a membrane will provide a high permeate flux with a low fouling potential [38]. In addition, an increase in pore size and high porosity of a membrane showed low contact angle values [39]. Authors have also stated that their experimental data showed the porosity factor as having the biggest influence on the surface contact angle. However, the current study found that the contact angle of the PLA-PEG membrane (76°) was reduced to 54° in the PLA-PEG/MgSiO_3_ membrane despite its high porosity and non-uniformity. This was due to the existence of free silicate species on the membrane surfaces upon the addition of MgSiO_3_. MgSiO_3_ acts as a dynamic hydrophilic material that favors the formation of the Si–OH group which makes the membrane more hydrophilic [40]. In addition, the presence of homopolymer PEG also improves the hydrophilicity and antifouling characteristic of the membrane [37].

### 3.2. Response Surface Methodology

Response surface methodology (RSM) was used to evaluate the influence of the parameters on output variables (response) and the optimum conditions for responses. The complete interactions between four parameters of lead removal using RSM, which include adsorbent mass, initial concentration, pH and temperature, were investigated by a few studies [26,41]. They discovered that the most significant parameters that could affect the adsorption performance were adsorbent mass and initial concentration of solution. This finding is also consistent with a later study which found the adsorption parameter at room temperature gave the highest removal efficiency of cationic dye [42]. This parameter suits the condition to be applied at the industrial scale. Therefore, the current work focuses on two significant parameters in producing the highest removal of MB dyes by using RSM, mainly mass of membrane (A) and initial concentration of MB solution (B). The effect of pH was investigated separately after obtaining the optimum condition for the adsorption process because MB dye is very sensitive to the pH of the solution. At low pH, MB dye tends to self-degrade, whereas at high pH MB dye will change color to saturated blue [43]. In this study, a total of 13 experiments using five-level central composite design (CCD) were carried out to verify the reliability models for optimization parameters that showed their interactions presented along with significant effects. The actual response values and predicted values for the experimental run are stated in Table 2.

#### 3.2.1. ANOVA Analysis

The parameters involved were statistically assessed though the analysis of variance (ANOVA). This analysis was necessary to test the significance and adequacy of the predicted models through the MB removal efficiency by membranes. Several model terms in ANOVA such as F-value, *p*-value, R-squared and lack of fit were evaluated and compared with experimental results for the fitting model [44]. Based on ANOVA analysis, the most suitable model that could explain the adsorption performance for these membranes was found to be the polynomial quadratic model. Table 3 and Table 4 summarized the analysis of variance (ANOVA) results of PLA-PEG and PLA-PEG/MgSiO_3_ membranes. Meanwhile, an empirical equation developed by the model correlated to the response (MB removal efficiency) and parameters (A, B)_,_ for both experimental results is as follows:

R% of PLA-PEG = + 30.42 + 12.01A − 8.94B − 7.34AB − 1.20A^2^ − 10.72B^2^ (3)

R% of PLA-PEG/MgSiO_3_ = + 86.36 + 12.72A − 11.14B + 6.30AB − 12.98A^2^ − 2.94B^2^ (4)

where A and B were the mass of membrane (mg) and initial concentration of MB solution (mg/L), respectively. The positive sign in these equations indicated a synergistic effect whilst the negative sign represented an antagonistic effect. It means that the increasing of parameter A gave a positive effect on MB removal and vice versa.

Next, based on Table 3 and Table 4, the membranes model displays statistically significant F-value, 62.67 and 51.43, respectively, with a *p*-value less than 0.0001. This indicates that only a 0.01% chance of “Model F-Value” could occur due to noise. As highlighted, values of the *p*-value show the probability of discarding an irrelevant hypothesis and values greater than 0.05 specify that the model terms are insignificant [45]. Hence, a large F-value and a *p*-value less than 0.05 indicate a significant model. Moreover, insignificant values of “Lack of Fit F-value” are desirable for the models to fit as it is a comparison between residual error and the pure error. The “Lack of Fit F-value” for both models were 0.54 and 0.38, which were insignificant relative to the pure error.

**Figure 5:**
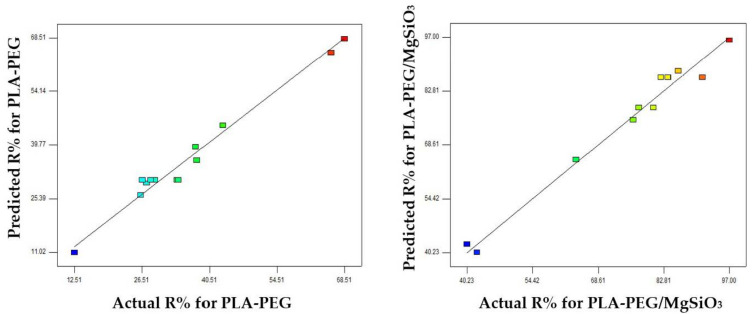
Diagnostic plot for actual and predicted of MB removal.

Another important principal part in ANOVA is the value of R-squared (R^2^). R^2^ presents the suitability of the model that fits with the experimental data to predict the response. It discloses the variability in percentage removal of MB and the degree of correlation between the response and parameters by the models [46]. The R^2^ for the PLA-PEG membrane was 0.9781 while for the PLA-PEG/MgSiO_3_ membrane was 0.9730. The regression model with R^2^ value above 0.9 is assumed to have a high correlation model [47]. Furthermore, an adequacy precision was used to measure the ratio of signal to noise, and the ratio should be greater than 4. As a result, both ratios indicate an adequate signal much higher than the desirable value, at 27.88 and 21.93, respectively. Figure 5 demonstrates a diagnostic plot between actual and predicted removal of MB efficiency. As can be observed, a good correlation can be seen through the data distribution that is close to the linear of each plot. Above all, these model terms revealed that this quadratic model was acceptable and fit with experimental data.

#### 3.2.2. Surface Plot and Contour

**Figure 6:**
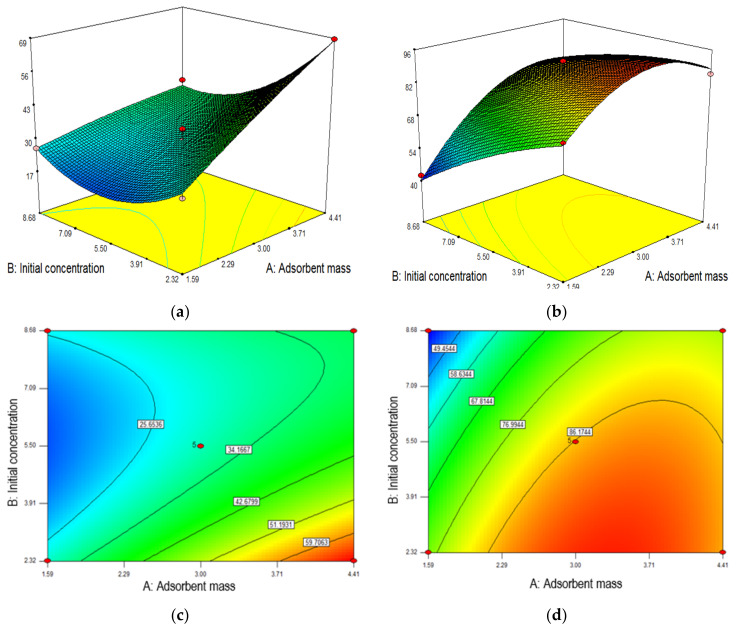
The 3D surface and contour plots for (**a**,**c**) PLA-PEG and (**b**,**d**) PLA-PEG/MgSiO_3_ membrane.

The performance of the adsorption process was interpreted by visualizing the 3D response surface and contour plot of the removal of MB dye, which helps reveal the contribution of factors A and B and an interaction between them [48]. Figure 6 illustrates 3D and contour plot for the optimization of the two parameters with their respective responses in the function of A, mass of membrane (adsorbent mass) and B, initial concentration of MB solution. The adsorption performance of the membranes was determined by the removal efficiency of the MB solution. Based on Figure 6a,b, it can be observed that the adsorption performance of PLA-PEG and PLA-PEG/MgSiO_3_ membranes increase with the increasing of mass of membrane up to 68% and 86%, respectively. This is due to that an increase in membrane surface area provides a greater number of adsorption sites available on the membrane surface [49,50]. More interestingly, PLA-PEG/MgSiO_3_ membrane displayed a better MB dye removal due to the presence of MgSiO_3_. This filler contained a Si-O group that enables the membranes to exhibit higher adsorption sites and a negatively charged surface [51]. Figure 6b also showed that 3 mg mass of membrane was sufficient to achieve maximum percentage removal, and further mass addition would not affect the adsorption performance since most of the dye molecules had become embedded to the surface sites.

The negative effect can be viewed upon increasing the initial concentration of MB. The decrease in adsorption performance of both membranes may be due to the saturation of adsorption sites on the membrane surface. At lower concentrations, the ratio of MB molecules available to the adsorption sites is low which results in a high percentage of removal. The increase of concentration of MB solution contributed to a higher ratio of MB molecules to the adsorption site. This phenomenon would saturate the adsorption sites in a short time, thus leaving a higher residual of MB dye in the solution [52]. However, as can be seen in Figure 6a, the removal of MB dye initially decreased along with the concentration increment before it rose after 5.5 mg/L. The increase of MB removal after 5.5 mg/L happened due to the large mass transfer driving force caused by the high initial concentration of MB [53]. Overall, it can be concluded that an increase in membrane amount gave a more positive effect than an increase in the initial concentration of MB.

#### 3.2.3. Optimization Using the Desirability Functions

The purpose of optimization was to identify the optimum condition for the highest removal efficiency of the MB solution. This test was performed by using numerical optimization through selecting a desirable function of parameters and response in RSM software. In this study, the parameters and the response were designed to be in given specific ranged values. The details of the optimum conditions are presented in Table 5. The finding revealed that the optimum conditions that resulted in the highest adsorption of MB for both membranes were 3 mg of mass of membrane with 5.5 ppm of the initial concentration of MB solution. It was convincing that these models were accepted and applicable as the desirability was 1.000.

### 3.3. Effect of pH on Adsorption Performance

**Figure 7:**
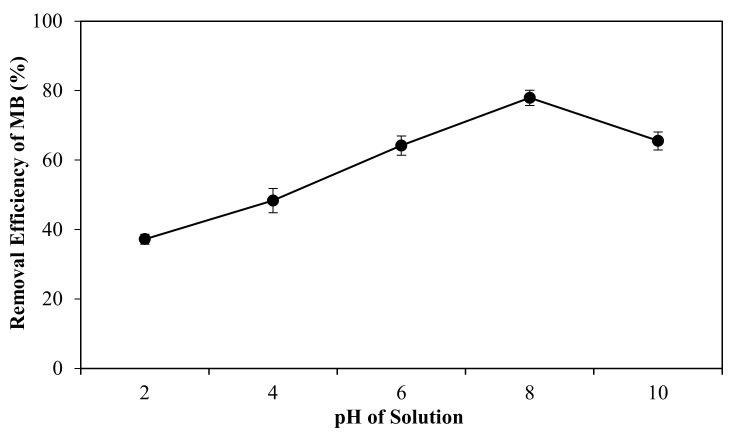
Effect of pH solution for adsorption performance.

PLA-PEG/MgSiO_3_ membrane with 3 mg of mass was chosen to study the effect of pH with the constant concentration of MB solution at 5.5 ppm. These constant parameters were obtained from the optimization condition using RSM software. The influence of pH as a medium was crucial to control the magnitude of electrostatic charges which are imparted by the ionized dye molecules. It is also needed as the pH of solution can ominously change the surface charges of the adsorption system [54]. Figure 7 showed the effect of pH solution ranging from 2 to 10. In general, the increase of pH increases the removal efficiency of MB dye. The removal efficiency of MB dye was elevated from 37% to 79% in the pH range of 2 to 8 and slowly lessened to 65% at pH 10. Recent research also suggested that a pH increase would adversely affect the adsorption removal for cationic dyes due to the increase in the number of negatively charged surface sites on the membrane [55]. This is because, at lower pH (pH ˂ 4), H^+^ from acidic medium will compete with cationic dye (MB^+^), thus limiting the adsorption of MB onto the negatively charged membrane. In addition, the large number of H^+^ ions in the solution can consequently enable Si–OH groups to change to Si–OH_2_^+^. This phenomenon would cause an electrostatic repulsion between Si–OH_2_^+^ and MB^+^ which also contributes to the lowering of MB removal. As the pH increases, the membrane surface becomes more negatively charged as the Si–OH groups presented in the membrane tend to change to Si–O^−^ [16]. This phenomenon will lead to stronger electrostatic interaction between the membrane and MB molecules. However, the decrease of adsorption performance at pH 10 corresponding to a higher isoelectric point would make the MB molecule surface become negatively charged and deprotonated, hence the electrostatic repulsion between the membrane and MB molecules is more likely to occur.

### 3.4. Ionic Strength

**Figure 8:**
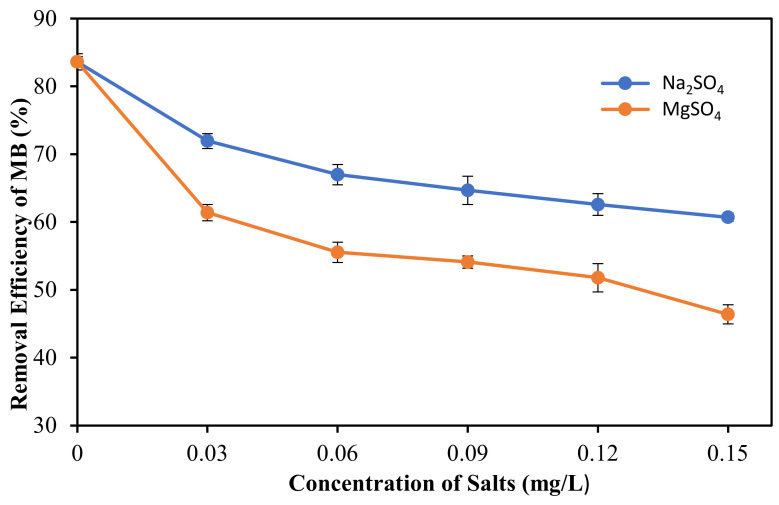
Removal efficiency of MB dye at different ionic strength using Na_2_SO_4_ and MgSO_4_ solution at pH = 6.65.

The effect of ionic strength towards the MB adsorption on the PLA-PEG/MgSiO_3_ membrane was studied using different salts concentrations ranging from 0 to 0.15 M. The salts used in this experiment were Na_2_SO_4_ and MgSO_4_ at the pH of distilled water (6.65). This test helps in revealing the adsorption mechanism involved during the adsorption process. Based on the results obtained, the increase in ionic strength decreases the removal efficiency of MB dye. Figure 8 shows a similar trend exhibited by Na_2_SO_4_ and MgSO_4_ solutions. This finding was consistent with past studies in which it was observed that the ionic strength of salts may affect the removal efficiency of cationic dye [56,57]. By using Na_2_SO_4_ solution, the removal efficiency of MB was reduced from 84% to 60% in the ionic strength of 0.15 mg/L due to the increase of Na^+^ amount in the solution, preventing MB from binding on the membrane surface. The increase of ionic strength leads to higher competition between MB^+^ and Na^+^ ions to adsorb to the surface. Another likely cause was the formation of ion pairs between MB^+^ and other anions (SO_4_^2−^) present in the solution that inhibit the activity of free MB^+^ ions. For MgSO_4_, there was a fluctuate drop due to the higher charge affinity of MgSO_4_ salt. Mg^2+^ displays stronger affinity than Na^+^/MB^+^ and gets adsorbed on the membrane [58]. As a result, the removal efficiency of MB dye was decreased significantly along with the increase of the ionic strength of the MgSO_4_ solution. From these results, it can be concluded that the removal efficiency of MB was dependent on the ionic strength of the solution. A previous study suggested that the adsorption system that is dependent on ionic strength mainly occurs via the ion-exchange mechanism at the negative site of adsorbent [59].

### 3.5. Adsorption Kinetics

**Figure 9:**
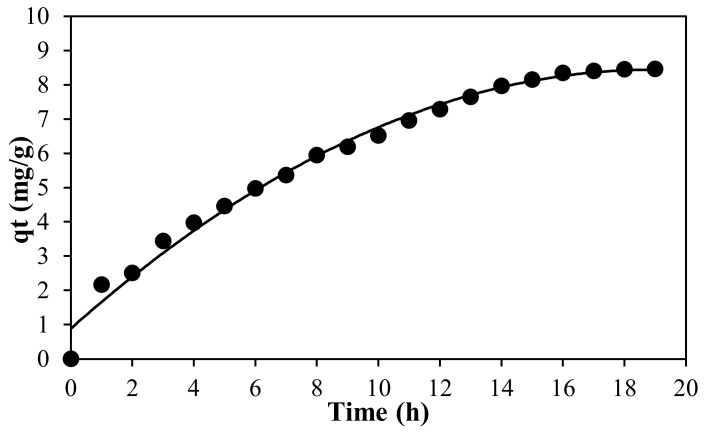
Adsorption capacity of MB.

Adsorption kinetics was performed to reveal the mechanism of adsorption, diffusion control and mass transfer. The experimental data were obtained through the function of adsorption capacity and the time consumed until it reached equilibrium state. In this present work, initial adsorption removal was increased rapidly, followed by a slow increase until equilibrium state was achieved after 19 h, as shown in Figure 9. This trend was consistent with the concept of mass transfer, where the high initial adsorption removal rate was due to large numbers of available adsorption sites, whereas the gradual increase of adsorption in time was ascribed to the resistance while reaching adsorption sites of the membrane [15].

There are two most common kinetic models used to examine the mechanism involved: the pseudo first order and pseudo second order models. These models can be expressed by the following integrated linear and non-linear forms:

Pseudo first order equation:

(5) $$\begin{array}{c} \mathrm{Linear}\;\mathrm{Form}\;\log\;(q_{e}-{\;q}_{t})=\log q_{e}-\frac{k_{1}}{2.303}t \end{array}$$

(6) $$\begin{array}{c} \mathrm{Non}-\mathrm{linear}\;\mathrm{Form}\;\mathrm{qt}=\mathrm{qe}-\left(1-\exp^{-k_{1}t}\right) \end{array}$$

**Figure 10:**
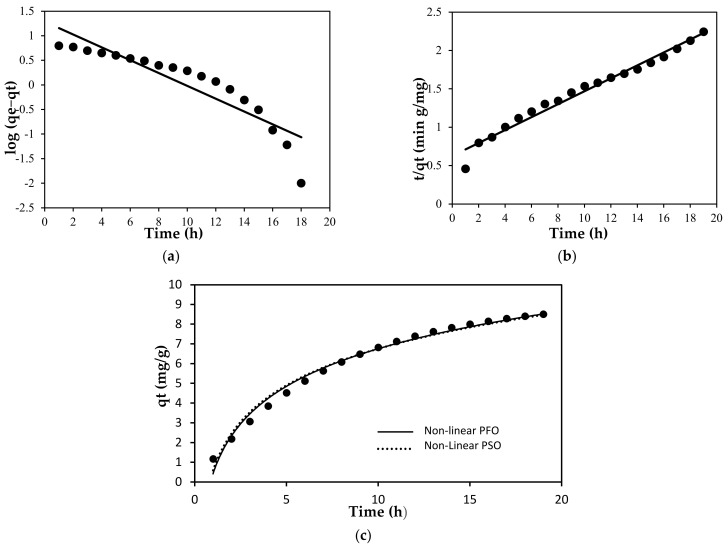
Adsorption Kinetic for (**a**) pseudo first order, (**b**) pseudo second order and (**c**) non-linear pseudo first order and non-linear pseudo second order.

Pseudo second order equation:

(7) $$\begin{array}{c} \mathrm{Linear}\;\mathrm{Form}\;\frac{t}{q_{t}}=\frac{1}{k_{2}q_{e}^{2}}+\frac{t}{q_{e}} \end{array}$$

(8) $$\begin{array}{c} \mathrm{Non}-\mathrm{linear}\;\mathrm{Form}\;\mathrm{qt}=\frac{k_{2}\cdot {\mathrm{qe}}^{2}\cdot t}{1+k_{2}\cdot {\mathrm{qe}}^{2}\cdot t} \end{array}$$

where q_e_ is the amount of dye adsorbed at equilibrium, q_t_ is the adsorbed amount at time t (mg/g), k_1_ is the rate constant (min^−1^) and k_2_ is the rate constant of pseudo second order (gmol^−1^ min^−1^). The data given in Table 6 present the parameter of adsorption kinetics models. The determination coefficient, R^2^ values (R^2^ = 0.9815, 0.9881) in both forms of pseudo second order model as seen in Figure 10, were higher than the pseudo first order model, which signifies a better adsorption process. Compared to the result obtained in both regression techniques (linear and non-linear forms), we noticed that the adsorption kinetics is favorable to the pseudo second order model with higher R^2^ values. This indicated that the adsorption of MB onto the PLA-PEG/MgSiO_3_ membrane was ruled by chemisorption interactions which involved valence forces through sharing or exchange of electron between the membrane and the MB molecules [60].

### 3.6. Adsorption Isotherms

Adsorption isotherm was necessary to study the adsorption behavior, prediction of the maximum adsorption and the affinity between membrane and adsorbate (MB dye). In this solid–liquid adsorption system, the distribution of dye molecules’ adsorption could be determined when it reached equilibrium state. The finding suggested that the adsorption performance was increased with the increase in the initial concentration of MB until it approached equilibrium. It is essential to identify the most fitting adsorption isotherm model by understanding the adsorption behavior. Out of several isotherm models available, there were two isotherm models that are frequently investigated, namely the Langmuir and Freundlich isotherms model. The Langmuir model presumes that the adsorption process occurs in a monolayer at specific homogeneous sites with a linearized and non-linear equation form below:

(9) $$\begin{array}{c} \mathrm{Linear}\;\mathrm{Form}\;\frac{C_{e}}{q_{e}}=\frac{1}{Q_{m}b}+\frac{C_{e}}{Q_{m}}\; \end{array}$$

(10) $$\begin{array}{c} \mathrm{Non}-\mathrm{linear}\;\mathrm{Form}\;\mathrm{qe}=\frac{Q_{m}\cdot \mathrm{Ce}}{1+b\cdot \mathrm{Ce}} \end{array}$$

where C_e_ is equilibrium concentration of MB solution, b is a Langmuir constant and Q_m_ represents a partial limiting adsorption maximum. The values of b and Q_m_ were calculated from the plot graph. On the other hand, the Freundlich model was proposed to describe the heterogeneous adsorption process which takes place through a multilayer adsorption with non-uniform distribution of adsorption heat and affinities. It is also known as non-ideal and reversible adsorption [61]. The Freundlich model can be expressed by the equation below, where K_F_ is the Freundlich constant and n is the affinity of the adsorbate to the membrane.

(11) $$\begin{array}{c} \mathrm{Linear}\;\mathrm{Form}\;\log q_{e}=\log K_{f}+\frac{1}{n}\log C_{e} \end{array}$$

(12) $$\begin{array}{c} \mathrm{Non}-\mathrm{linear}\;\mathrm{Form}\;\mathrm{qe}=K_{F}\cdot {\;\mathrm{Ce}}^{1/n} \end{array}$$

Table 7 shows the calculated determination coefficients (R^2^) and model parameters of the Langmuir and Freundlich isotherms model. From the result obtained, the experimental data fitted better with Freundlich model (R^2^ = 0.9796, 0.9028) in comparison with Langmuir. This indicated that the adsorption process that occurred was reversible and the n value of the Freundlich equation could give an indication on the favorability of sorption. It is generally stated that the values of n in the range of 1 to 10 represent good adsorption.

### 3.7. Reusability and Regeneration Study

**Figure 11:**
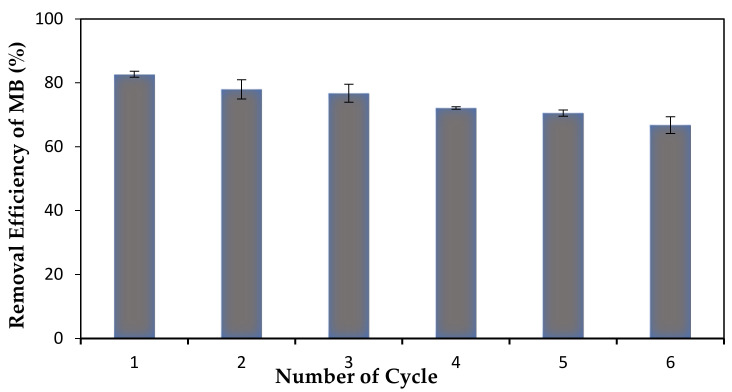
The cycles of reusability and regeneration for PLA-PEG/MgSiO_3_ membrane.

The stability and efficiency of this membrane upon reuse was investigated through reusability and regeneration study. It helps in determining the significance of adsorption performance upon repeating and restoration of its original characteristics. Regeneration can be defined as the recovery of the active sites after the adsorption process which took place by removing the dye molecules with acidic, alkaline or deionized water [58]. The regeneration of active sites upon repeated use is proportional to its stability. As shown in Figure 11, this membrane presented excellent adsorption performance as well as desorption process even after six cycles. Throughout these six cycles, PLA-PEG/MgSiO_3_ still displayed a good regeneration characteristic even though the removal efficiency of MB dye was decreased from 84% to 66%. This was due to the possibility of an intramolecular breaking bond and that not all the dyes’ molecules were completely desorbed after being immersed in methanol solution during the desorption process [62]. Additionally, the findings for this study have a good agreement with the results obtained in adsorption isotherms which fit the Freundlich model. The Freundlich model exhibited a reversible adsorption process, which could explain this entire adsorption system.

### 3.8. Adsorption Mechanism

**Figure 12:**
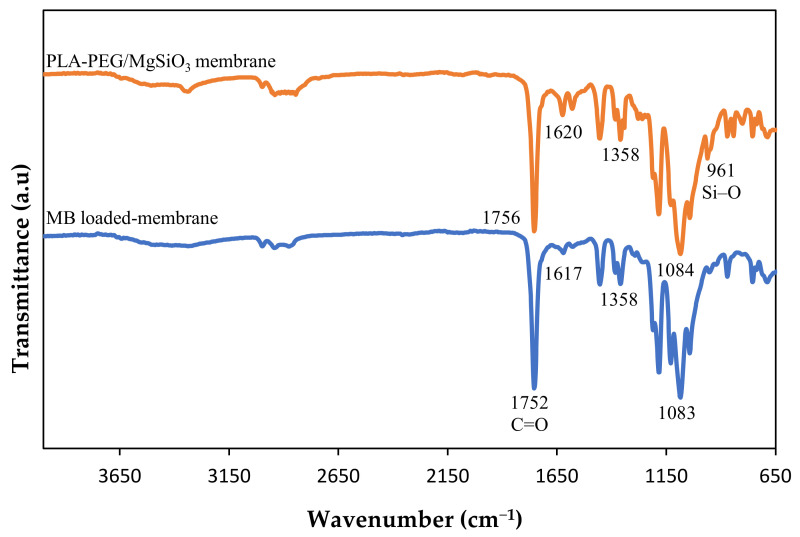
FTIR spectra of PLA-PEG/MgSiO_3_ before and after adsorption.

The most obvious finding to emerge from this study is the types of interactions that occurred during adsorption process, as understood from the results obtained from the experiments. In this case, the interaction mainly occurred at two sites, which are at the membrane’s surface and the surface of the MgSiO_3_ filler. From the results of FTIR shown in Figure 12, some of the functional groups shifted after the MB dye was loaded to the membrane. The absorption bands of N–H at 1620 cm^−1^ migrated to 1617 cm^−1^ which may correspond to the formation of a hydrogen bond between the MB dye and PLA-PEG matrix [16]. Next, the change of C=O groups from 1756 cm^−1^ to 1752 cm^−1^ may have also been contributed to by dipole moment interaction [63].

**Figure 13:**
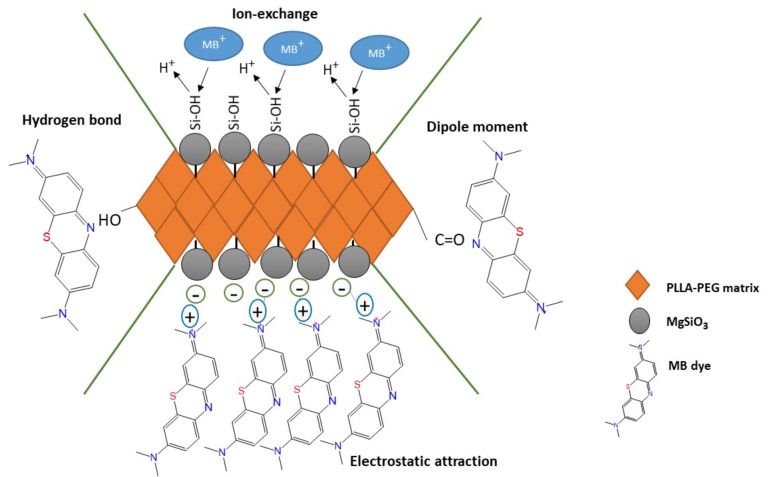
Proposed adsorption mechanism for the removal efficiency of MB dye.

Moreover, this adsorption system followed the pseudo second order model that suggests that the removal of MB dye was ruled by chemisorption interaction. This can be proven with the results of the pH effect that could be related to the electrostatic interaction. The removal of MB dye was low in acidic medium while in the alkali medium, the removal efficiency of MB dye increased. Other than that, the effect of ionic strength also seems to offer a significant contribution to the study of the adsorption mechanism. The increasing of concentration of salts significantly decreased the removal efficiency of MB dye as MB^+^ must compete with Na^+^/Mg^2+^ ions to be adsorbed at active sites of the membrane. Previous literature has reported that ion-exchange was the key mechanism for the MB removal due to the effect of ionic strength [64]. Therefore, we can conclude that the main mechanisms involved in removing MB dye by PLA-PEG/MgSiO_3_ were electrostatic attraction and ion-exchange mechanism, along with other interactions such as hydrogen bond and dipole moment. Figure 13 shows the suggested adsorption mechanism for this research work.

## 4. Conclusions

The present work was designed to determine the removal efficiency of MB using PLA-PEG and PLA-PEG/MgSiO_3_ membranes. In term of membrane performance, PLA-PEG/MgSiO_3_ showed a better tensile strength. It also exhibited a more hydrophilic character even though PLA-PEG displayed high porosity and larger surface pore size due to the existence of silicate groups. These results revealed that the addition of filler results in a major contribution to the contact angle. Optimization of parameters in removing MB dye using RSM showed that the PLA-PEG/MgSiO_3_ membrane has better removal activity of MB dye at 86.36%, higher than PLA-PEG membrane (30.42%). The optimum condition was obtained at 3 mg of the mass of membrane with 5.5 ppm of MB solution initial concentration. Then, 3 mg of PLA-PEG/MgSiO_3_ membrane and 5.5 ppm of MB initial concentration was chosen to examine the effect of pH and ionic strength, adsorption mechanism and behavior. The adsorption performance fitted well with Freundlich isotherm model and pseudo second order for adsorption of kinetic with R^2^ showing 0.9796 and 0.9815, respectively. The main mechanism of adsorption was believed to be electrostatic attraction and ion-exchange mechanism since the membrane was dependent upon the effect of pH and ionic strength. Overall, the PLA-PEG/MgSiO_3_ membrane displayed good membrane properties and could be reused for up to six cycles.

## Figures and Tables

**Table 1:** Ranges and levels of independent variables.

Independent Variables	Range and Level				
	−α	−1	0	+1	+α
Mass of membrane, A (mg)	1.00	1.59	3.00	4.41	5.00
Initial concentration of MB, B (mg/L)	1.00	2.32	5.50	8.68	10.0

**Table 2:** Actual and predicted values of methylene blue (MB) removal efficiency (%).

No.	A (mg)	B (ppm)	PLA-PEG		PLA-PEG/MgSiO_3_	
			Actual Value	Predicted Value	Actual Value	Predicted Value
1	1.59	2.32	27.52	29.52	76.15	75.16
2	4.41	2.32	68.51	68.22	85.94	88.00
3	1.59	8.68	26.28	26.32	42.38	40.27
4	4.41	8.68	37.91	35.66	77.38	78.32
5	1.00	5.50	12.51	11.10	40.23	42.41
6	5.00	5.50	43.25	44.99	80.52	78.39
7	3.00	1.00	65.75	64.49	97.00	96.23
8	3.00	10.500	37.69	39.20	63.91	64.72
9	3.00	5.50	33.80	30.42	83.74	86.36
10	3.00	5.50	29.23	30.42	83.66	86.36
11	3.00	5.50	34.12	30.42	91.14	86.36
12	3.00	5.50	26.59	30.42	82.13	86.36
13	3.00	5.50	28.34	30.42	91.14	86.36

**Table 3:** ANOVA analysis for quadratic model of PLA-PEG.

Source	Sum of Squares	df	Mean Square	F-Value	*p*-Value (Prob > F)
Model	2855.88	5	571.18	62.67	<0.0001
X_1_	1154.23	1	1154.23	128.63	<0.0001
X_2_	639.44	1	639.44	70.15	<0.0001
X_1_X_2_	215.50	1	215.50	23.64	0.0018
X_1_^2^	10.09	1	10.9	1.11	0.3277
X_2_^2^	798.80	1	798.80	87.64	<0.0001
Residual	63.80	7	9.11	-	-
Lack of Fit	18.28	3	6.09	0.54	0.6826
Pure Error	45.53	4	11.38	-	-
Total	2919.69	12	-	-	-

**Table 4:** ANOVA analysis for quadratic model of PLA-PEG/MgSiO_3_.

Source	Sum of Squares	df	Mean Square	F-Value	*p*-Value (Prob > F)
Model	3629.79	5	725.96	51.43	<0.0001
X_1_	1294.61	1	1294.61	91.71	<0.0001
X_2_	992.94	1	992.94	70.34	<0.0001
X_1_X_2_	158.89	1	158.89	11.26	0.0122
X_1_^2^	1172.33	1	1172.33	83.05	<0.0001
X_2_^2^	60.20	1	60.20	4.26	0.0778
Residual	98.81	7	14.12	-	-
Lack of Fit	21.07	3	7.02	0.38	0.7865
Pure Error	77.74	4	19.44	-	-
Total	3728.60	12	-	-	-

**Table 5:** Optimum condition for removal efficiency of MB dye by membranes.

Membrane	Desirability	Parameter		Removal Efficiency of MB Solution		
		Mass of Membrane	Initial Concentration of MB	Actual	Predicted	Error
PLA-PEG	1	3 mg	5.5 ppm	30.42	30.416	0.004
PLA-PEG/MgSiO_3_	1	3 mg	5.5 ppm	86.36	86.362	0.002

**Table 6:** Kinetics parameter for adsorption performance.

Form	Plot	Parameter	Values
Pseudo first order			
Linear	C_e_/q_e_ vs. C_e_	K_1_	1.3393
		q_e_	5.066 × 10^−3^
		R^2^	0.8194
Non-linear	q_t_ vs. t	K_1_	0.1349
		q_e_	9.2058
		R^2^	0.9871
**Pseudo second order**			
Linear	Log q_e_ vs. log C_e_	K_2_	0.002479
		q_e_	11.14
		R^2^	0.9815
Non-linear	q_t_ vs. t	K_1_	0.00932
		q_e_	12.5106
		R^2^	0.9881

**Table 7:** Isotherms parameter of Langmuir and Freundlich models.

Form	Plot	Parameter	Values
Langmuir model			
Linear	C_e_/q_e_ vs. C_e_	Q_m_	13.8696
		b	0.1415
		R^2^	0.8360
Non-linear	q_e_ vs. C_e_	Q_m_	25.1502
		b	0.1196
		R^2^	0.8807
**Freundlich model**			
Linear	Log q_e_ vs. log C_e_	1/n	0.7637
		K_F_	1.6866
		R^2^	0.9796
Non-linear	q_e_ vs. C_e_	1/n	0.6346
		K_F_	3.3324
		R^2^	0.9028

## Data Availability

Not applicable.
